# A History of Innovation: Tracing the Evolution of Imaging Modalities for the Preoperative Planning of Microsurgical Breast Reconstruction

**DOI:** 10.3390/jcm12165246

**Published:** 2023-08-11

**Authors:** Jevan Cevik, Ishith Seth, David J. Hunter-Smith, Warren M. Rozen

**Affiliations:** 1 Department of Plastic and Reconstructive Surgery, Peninsula Health, Frankston, VIC 3199, Australia; 2 Peninsula Clinical School, Central Clinical School, Faculty of Medicine, Monash University, Frankston, VIC 3199, Australia

**Keywords:** history, breast reconstruction, innovation, preoperative planning

## Abstract

Breast reconstruction is an essential component in the multidisciplinary management of breast cancer patients. Over the years, preoperative planning has played a pivotal role in assisting surgeons in planning operative decisions prior to the day of surgery. The evolution of preoperative planning can be traced back to the introduction of modalities such as ultrasound and colour duplex ultrasonography, enabling surgeons to evaluate the donor site’s vasculature and thereby plan operations more accurately. However, the limitations of these techniques paved the way for the implementation of modern three-dimensional imaging technologies. With the advancements in 3D imaging, including computed tomography and magnetic resonance imaging, surgeons gained the ability to obtain detailed anatomical information. Moreover, numerous adjuncts have been developed to aid in the planning process. The integration of 3D-printing technologies has made significant contributions, enabling surgeons to create complex haptic models of the underlying anatomy. Direct infrared thermography provides a non-invasive, visual assessment of abdominal wall vascular physiology. Additionally, augmented reality technologies are poised to reshape surgical planning by providing an immersive and interactive environment for surgeons to visualize and manipulate 3D reconstructions. Still, the future of preoperative planning in breast reconstruction holds immense promise. Most recently, artificial intelligence algorithms, utilising machine learning and deep learning techniques, have the potential to automate and enhance preoperative planning processes. This review provides a comprehensive assessment of the history of innovation in preoperative planning for breast reconstruction, while also outlining key future directions, and the impact of artificial intelligence in this field.

## 1. Introduction

Breast reconstruction surgery has a long history, culminating in it becoming a vital part of the treatment of breast cancer in the modern day [1]. Breast reconstruction aims to restore the form and self-confidence of women who have undergone oncological resection of the breast [2]. Significant advancements in breast reconstruction over the past century have substantially improved the ability to reconstruct the breast to maintain natural symmetry, aesthetics, and sensation.

Early techniques focused primarily on the use of prosthetic implants for aesthetic reconstruction, yet, following significant breakthroughs in our understanding of vascular anatomy and microsurgical tissue transfer, a resurgence of autologous breast reconstruction took place [3,4,5,6,7,8]. Numerous donor sites have been reported, yet, of the options available, the abdominal wall became one of the more popular sites for autologous tissue given the frequent presence of excess soft tissue and its ability to closely resemble native breast tissue [9,10]. In 1994, Allen and Treece introduced the deep inferior epigastric artery perforator (DIEP) flap, revolutionising the field of breast reconstruction [9]. Unlike traditional muscle-based myocutaneous flaps, the DIEP flap spares the rectus abdominis muscle by utilising only the skin and fat tissue, resulting in reduced donor site morbidity and improved abdominal wall function. However, perforator-based flaps of the abdominal wall, such as the DIEP flap, exhibit significant variability in their vascular supply, confronting surgeons with a new challenge and necessitating precise preoperative planning to ensure the viability and success of the reconstructive procedure [11,12,13,14].

The complexity of vascular anatomy and the need for meticulous surgical planning underscored the importance of preoperative imaging in breast reconstruction. This review details the history and current state of preoperative imaging in breast reconstruction, and discusses the impact of artificial intelligence in this field.

## 2. Methodology

Two independent authors (JC and IS) searched for relevant studies on PubMed, Google Scholar, Web of Science, and Scopus databases from January 1901 to June 2023. The search terms consisted of different combinations of “history”, “breast”, “breast reconstruction”, “breast reconstruction surgery”, “imaging”, “medical imaging”, “ultrasound”, “ct”, “MRI”, “thermography”, “three-dimensional printing”, “3D”, “preoperative”, “preoperative planning”, “preoperative care”, “artificial intelligence” and “innovation”. The search strategy was designed to encapsulate all studies that discussed the history and innovation of imaging in breast surgery. In addition, the reference lists of previous reviews and the relevant literature were manually checked. Emphasis was placed on the inclusion of studies that discussed the development, utilisation, and effectiveness of imaging modalities for the preoperative planning of breast reconstruction, presented new advances in imaging technology, included direct comparisons between different imaging modalities or discussed shifts in surgical/preoperative planning practice. All studies were available in English or could be accurately translated. Conversely, studies were excluded if they did not specifically address imaging techniques for DIEP flap planning, presented anecdotal or case-based evidence, were published in non-peer-reviewed journals, were opinion pieces without original research, primarily discussed postoperative imaging or intra-operative imaging methods, or were studies with inaccessible full texts.

## 3. Early Techniques

With the advent of perforator-based flaps, the visualisation and identification of perforators was initially performed using Doppler ultrasound. First introduced to the medical field in the 1950s, Doppler ultrasound was primarily used for cardiovascular applications, exploring the function of the heart and flow of blood in the human body [15,16,17]. By the 1970s, the medical field began recognising its potential applications in other areas, including reconstructive microsurgery [18]. As surgeons realised the importance of understanding the vascular architecture for successful breast reconstruction, Doppler ultrasound emerged as a non-invasive, real-time imaging technique with which to identify perforators and map vascular anatomy, enabling more accurate surgical planning [19,20]. 

As the only available option for surgeons early on, Doppler ultrasound showed promise as a fast, readily available, and simple method of perforator identification. Early research suggested that Doppler ultrasound had encouraging accuracy in detecting perforators preoperatively [19,20,21,22]. However, while it offers some insight into the vascular network, it does not provide a comprehensive three-dimensional map, differs in results depending on the user, and can occasionally miss smaller vessels or misinterpret the flow of superficial main vessels as perforators [23,24,25]. This is highlighted in studies that reveal the Doppler probe’s occasional inaccuracies when juxtaposed with surgical observations or more modern imaging techniques, particularly with regard to smaller, deeply located vessels [26,27,28]. Nevertheless, ultrasound remains a popular and widely utilised preoperative imaging modality for breast reconstruction planning [29,30].

Building upon Doppler ultrasound, colour duplex ultrasonography evolved to offer even further-improved visualisation. Introduced in the late 1970s, this technology combines the principles of Doppler ultrasound with conventional imaging, providing a two-dimensional anatomical representation of structures while simultaneously mapping the flow of blood [31,32,33]. The colour-coding adds an additional layer of information, distinguishing between vessels with blood flowing towards and away from the probe, and providing an immediate, visual understanding of the vascular territory [34,35,36]. Colour duplex ultrasonography improved the precision and accuracy of preoperative planning, proving accurate in identifying perforating vessels and in-fact exhibiting greater precision when compared with the standard Doppler ultrasound [37,38,39,40,41,42]. Some have even suggested that colour duplex ultrasonography may rival modalities such as computed tomographic angiography in its accuracy of perforator mapping [43]. Nevertheless, while marking another step forward in the realm of preoperative imaging of breast reconstruction, colour duplex ultrasonography requires expertise in assessment and suffers from many of the same limitations that afflict Doppler ultrasound, leaving much of the vascular territory under-represented.

Despite their limitations, Doppler ultrasound and colour duplex ultrasonography remain integral components of the preoperative planning arsenal.

## 4. Emergence of Advanced Imaging Modalities

One of the largest and most influential advances in the preoperative planning of breast reconstruction was the introduction of computed tomography angiography (CTA) to this field. CTA, first introduced in the early 1990s, utilizes ionising radiation in combination with intravenously administered contrast media to obtain high-resolution images of the body’s vascular system [44,45]. As CTA became more refined over the decades, its utilisation in preoperative imaging for breast reconstruction became more evident [46,47,48]. In particular, it has proven indispensable for autologous breast reconstructions using perforator flaps such as the DIEP flap, where it aids in visualising and mapping the intricate network of perforator vessels [10,49,50,51,52,53]. In the current day, CTA has become the gold-standard option for preoperative planning prior to surgery. 

**Figure 1:**
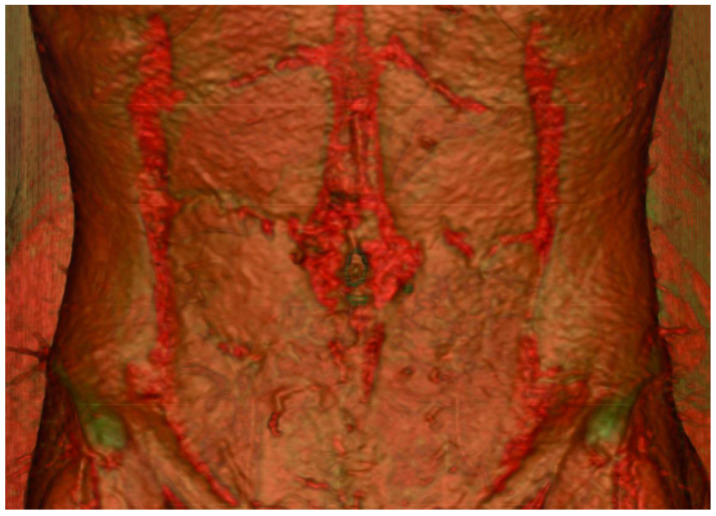
Three-dimensional reconstruction of the abdominal wall displaying the perforating vessels originating from the deep inferior epigastric artery.

**Figure 2:**
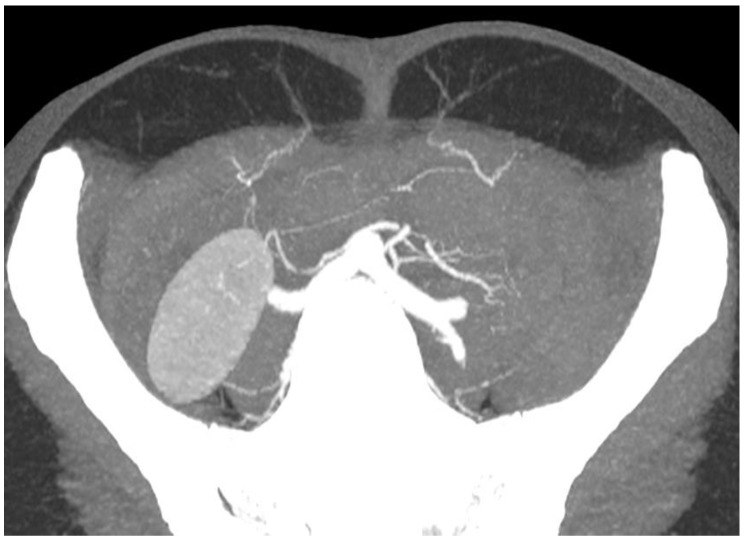
Axial CTA slice of the abdomen with maximum-intensity projection applied displaying the vasculature of the anterior abdominal wall and the intramuscular course of the medial row perforators.

CTA’s utility lies in its ability to provide a detailed three-dimensional visualisation of the vasculature. It has become readily available in most areas, remains relatively cheap and generates consistent images regardless of the observer. Via specialised scanning protocols and advanced rendering software, accurate post-processing reconstructions of real anatomical structures can be performed (Figure 1). Furthermore, the use of maximum-intensity projection techniques can help delineate the accurate intramuscular course of perforators to guide intra-operative dissection (Figure 2). 

CTA has several benefits that differentiate it from its predecessors of Doppler ultrasound or colour duplex ultrasonography. In comparison to these former imaging modalities, it has been shown to be more accurate, have a shorter scanning time, display smaller-diameter vessels with greater detail and provide information of the entire vascular tree, including the intramuscular and subcutaneous course of perforating vessels [27,54,55]. However, CTA has additional limitations not present in earlier imaging techniques, particularly the use of ionising radiation which can have a cumulative effect across multiple CT scans significantly increasing the risk of cancer and other diseases. Furthermore, the reliance on iodinated contrast is problematic, given that it is contraindicated in patients with renal impairment. Despite these limitations, numerous studies have displayed the benefits of utilising CTA preoperatively on several operative and postoperative factors including shorter operative times and a reduced length of hospital admission [51,56,57,58]. While some studies suggest a positive impact on postoperative complications such as flap failure, fat necrosis or donor site morbidity, this remains debated [59,60,61,62]. For instance, a recent randomised control trial by Colakoglu et al. in 2022 demonstrated shorter flap harvest and overall operative times when CTA was used preoperatively but no significant difference in overall complication rate [63]. Nevertheless, the potential economic benefits of a shorter operative duration and hospital admission are appealing [64].

Advancements in preoperative imaging for breast reconstruction did not stop with CTA. Magnetic resonance imaging (MRI), developed in the 1970s, was introduced for the identification of abdominal perforators by Ahn et al. in 1994 [65,66]. Unlike CTA, MRI uses a strong magnetic field and radio waves to produce detailed images of blood vessels without the use of ionising radiation, preventing potential harm to patients. However, MRI alone produces relatively low-resolution images, yet, when supplemented with contrast material such as gadolinium, can produce detail rivalling that of CTA [67]. This technique, named magnetic resonance angiography (MRA), emerged as another ground-breaking imaging technology in the late 1980s [67,68,69]. Since its inception, MRA has seen increasing application in preoperative planning for breast reconstruction [70,71,72,73]. As with CTA, MRA provides valuable information about the vascular network, allowing for the precise planning of flap-based reconstruction. Moreover, MRA’s ability to offer high-quality soft tissue contrast and its non-reliance on ionising radiation or iodinated contrast make it an attractive option in certain clinical scenarios. Studies have shown a relative equivalence between CTA and MRA in mapping the arterial perforators of the deep inferior epigastric artery (DIEA) [74,75,76]. Furthermore, like CTA, studies suggest that the use of MRA preoperatively may improve surgical outcomes postoperatively [74,77]. However, MRA remains significantly less available than CTA and more expensive, making access to this imaging modality challenging [78]. Moreover, it is more susceptible to motion artefacts and may provide less detail for vessels smaller in diameter [78,79]. Additionally, it requires a longer scanning time and has other contraindications such as internal metal-ware, certain implantable defibrillators, and patient claustrophobia (Table 1) [67,80]. Furthermore, there is a risk of allergic reaction to the gadolinium-based contrast agents. Moreover, gadolinium has been linked to nephrogenic systemic fibrosis in those with severe kidney dysfunction [81]. There have also been reports of gadolinium deposits in the brain and other tissues, but the long-term effects of these deposits are not well-understood [81]. Currently, these barriers have limited the widespread use of MRA; however, as technology continues to advance it could replace CTA as the gold-standard imaging modality.

## 5. Development of Imaging Adjuncts

### 5.1. Direct Infrared Thermography (DIRT)

The field of preoperative planning in breast reconstruction has seen the emergence of numerous technological adjuncts that augment the process of preoperative imaging. One such technique is direct infrared thermography (DIRT). 

**Figure 3:**
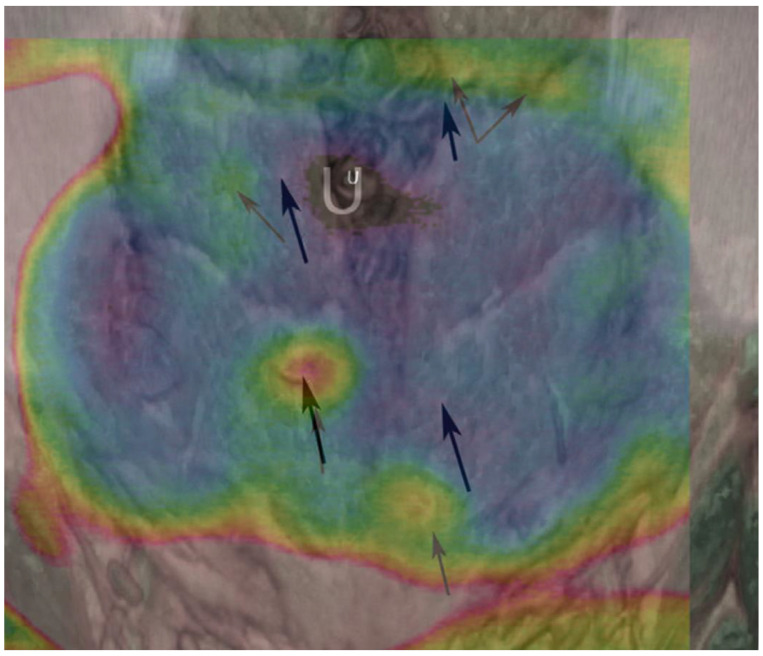
Direct infrared thermographic image overlaid onto a 3D-volume-rendered reconstruction of a CTA scan showing the correlation between hotspots on the thermographic display and the location of perforators (arrows) (reproduced with permission from Whitaker et al., 2012 [86]).

DIRT has a history that reaches back to the 1950s [82], with its main function being to measure and visualize the distribution of heat across the body’s surface, and it has found a significant role in microvascular free flap reconstruction with some of its earliest applications being described by Theuvenet et al. in 1986 [83]. Since then, DIRT has grown in popularity and its use in autologous breast reconstruction has increased [84,85,86,87]. The mechanism of DIRT utilises infrared cameras to detect the infrared radiation emitted from the skin, then convert this into a colour-coded visual map, displaying temperature variations. In a preoperative setting, it allows surgeons to assess areas of ‘hot spots’ which indicate the location of underlying perforators (Figure 3). The application of DIRT is typically dynamic; it begins with a “cold challenge” where the skin is cooled, and then the infrared camera monitors the pattern of subsequent reheating. 

The benefits of DIRT include its non-invasiveness, easy reproducibility and interpretability, and real-time dynamic feedback. It is also an imaging technique that is available throughout all stages of the surgical procedure including pre, peri and postoperatively. This allows for the additional benefit of not only helping with the planning of the flap but also as an adjunct to assess flap perfusion intraoperatively and postoperatively [88,89,90,91]. However, its limitations are substantial too. It is sensitive to environmental conditions and the topography of the body surface, which may lead to misinterpretation [87,92]. Additionally, DIRT only provides superficial heat data, not supplying information about deeper structures [87,92]. Furthermore, it provides little to no understanding of the structure of the underlying vasculature, only providing insights into the physiology of blood flow [87,92]. This makes it significantly less effective than traditional CTA or MRA at creating a morphological map of the vascular architecture prior to surgery. Despite these limitations, DIRT remains a safe, low-cost, and effective imaging adjunct that can augment the process of preoperative planning in breast reconstruction. 

In the modern day, DIRT has advanced to the point where infrared cameras have been developed that can simply connect to the everyday smartphone. Hardwicke et al. in 2016 demonstrated that perforators of the abdomen and thigh can be effectively and accurately localised using one such smartphone-compatible camera—Flir One [93]. This camera costs just over 200 USD and is easily portable given its small size. In their study, Hardwicke et al. demonstrated that this camera displays similar accuracy in mapping the perforators of the abdomen and thigh in comparison with that of conventional Doppler ultrasound [93]. Moreover, two years later, Pereira et al. in 2018 compared the accuracy of Flir One to that of CTA in identifying perforators of the anterolateral thigh [94]. They found that, amazingly, the Flir One infrared camera had a sensitivity of 100% and specificity of 98% [94].

### 5.2. Three-Dimensional Printing

**Figure 4:**
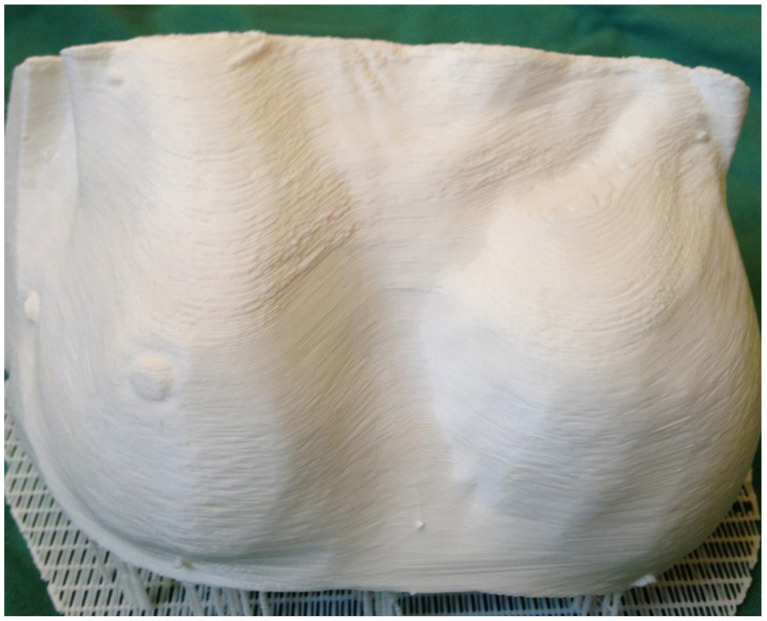
Three-dimensionally printed model of the breast for the preoperative planning of breast reconstruction (reproduced with permission from Chae et al., 2014 [103]).

Another technological adjunct in the armament of surgeons when planning breast reconstruction preoperatively is three-dimensional (3D) printing. This innovative technology has expanded rapidly since its inception in 1986 with applications across numerous medical disciplines [95]. In the preoperative planning of breast reconstruction, it enables the creation of physical models from digital files, typically derived from a patient’s CT or MRI scans. In 2011, Melchels et al. first introduced 3D printing to the field of breast reconstruction by constructing a model of a patient’s breast to aid in its reconstruction [96]. By utilising 3D-printed models (Figure 4), surgeons gain the ability to visualize, touch, and even simulate the surgical process, again leading to a more comprehensive understanding of the patient’s unique anatomy and specific requirements. The use of 3D-printed models has been shown in other surgical fields to reduce operative times and increase surgical accuracy [97,98]. In breast reconstruction, it has been suggested that it facilitates and makes easier the dissection of the intramuscular course of perforators [99]. In addition, with 3D-printed models, patients can physically interact with a tangible representation of their anatomy, facilitating a clearer understanding of the procedure and its potential outcomes [100,101]. This is potentially helpful in breast reconstruction where it may help alleviate anxiety, improve communication between the surgeon and the patient, and enable more informed decision-making. Yet, there are some limitations to consider. One is the costs associated with acquiring 3D printers and the materials required for printing, although this cost is seeing a rapid decline [102]. Additionally, the production of 3D-printed models can be time-consuming, as it involves data processing, model design, and printing [102].

### 5.3. Augmented Reality

**Figure 5:**
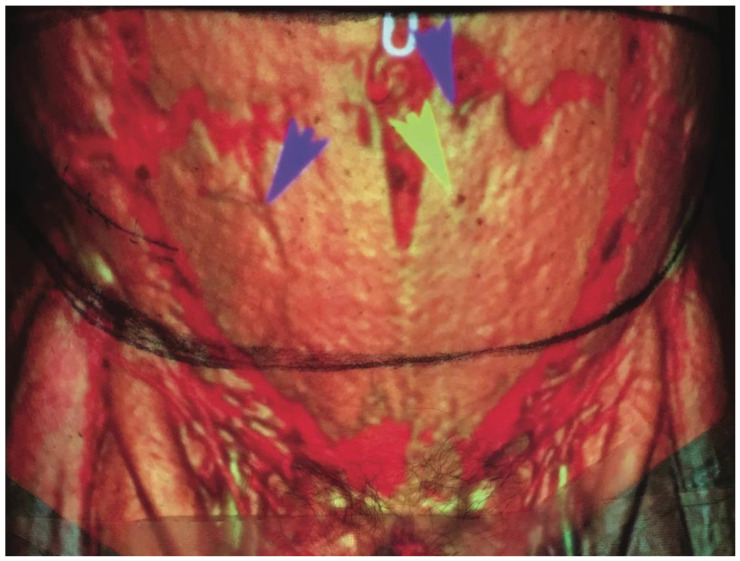
Augmented reality volume-rendered CTA projection overlaid onto patient’s anterior abdominal wall intra-operatively identifying the location of perforators with arrows (reproduced with permission from Phan et al., 2022 [110]).

Augmented reality (AR) is a recent addition to the surgical planning field, offering a means to visualize anatomical structures superimposed on the patient’s body in real-time (Figure 5). It integrates computer-generated images with the surgeon’s view of the patient, making the unseen seen. Like other advances in preoperative imaging, the benefits include improved anatomical understanding, intra-operative guidance, potentially shorter surgical times and assistance with surgical education [104,105,106]. In the realm of breast reconstruction, wearable augmented reality devices such as HoloLens™ (Redmond, WA, USA) have been demonstrated to offer surgeons the ability to visualise perforators and anatomy before and during an operation [107,108]. However, AR is not without its downsides. Firstly, there is the cost of AR equipment, and secondly, there is a learning curve for surgeons unfamiliar with the technology [109].

### 5.4. Contrast-Enhanced Ultrasound

The field of preoperative planning in reconstructive surgery has seen a resurgence of older methods in recent years. For instance, contrast-enhanced ultrasound, as described by Su et al. in 2013, has emerged as a novel technique, building upon the original technology [111]. The addition of intravenous ultrasound contrast agents provides additional reflective material in the vasculature and thereby increases the accuracy of the imaging modality in identifying perforators [111]. This technology has been shown to have remarkable sensitivity and specificity in identifying perforators in both anterolateral thigh flaps and, more recently, DIEP flaps [111,112,113]. Furthermore, this technology can be reconstructed into 3D images, providing a more comprehensive visualisation of vascular anatomy, assisting surgeons in the further optimisation of surgical planning [111].

## 6. Artificial Intelligence in Pre-Operative Planning

Artificial intelligence (AI) represents the most cutting-edge technology being integrated into pre-operative planning. AI describes a system that allows computers to display human-like intelligence. Machine learning (ML), a branch of AI, has seen applications in multiple areas of medical imaging [114]. These models detect certain features from images, such as textures or shapes, and apply statistical formulae to detect patterns that indicate anatomical structures, diseases, or conditions. A subset of ML, deep learning (DL), is also often applied to medical imaging. DL models, built to resemble the human structure and the function of the human brain, consist of interconnected “neurons” [115,116]. Initially, the model’s parameters are randomly set, and it generates predictions for a set of training images. These predictions are then compared to the known true identities of the images, and the model’s parameters are adjusted based on any errors made in judgement. After repetition, the model learns and can make predictions on unfamiliar, novel images.

AI has had a wide impact in the field of plastic surgery, with evidence supporting its use for a multitude of various applications. For instance, O’Neill et al. in 2020 demonstrated the use of a ML algorithm at identifying those at high risk of experiencing flap failure in their cohort [117]. Similarly, an AI algorithm has been shown to effectively predict the development of surgical site infections after free flap reconstruction [118]. These studies outline the potential for AI to aid in our ability to identify patients who are at high risk of developing postoperative complications and improve our ability to generate personalised surgical plans for such patients. 

Moreover, AI has been shown to offer significant benefits in the field of preoperative imaging. The traditional process of reporting preoperative imaging such as CTA scans remains a labour-intensive process that can often be challenging and requires specific expertise [10,50]. AI offers multiple possible benefits: potentially superior predictive accuracy, time savings for clinicians, and greater reliability. Mavioso et al. in 2020 demonstrated some of these benefits in their study analysing the efficacy of a semi-automatic model in identifying perforators in the preoperative planning of a DIEP flap [119,120]. They found that the AI algorithm resulted in approximately 80 h being saved for the 40 patients included in the study [119]. Additionally, Saxena et al. in 2022 created a deep learning model that could autonomously segment vessels in artificial images of vasculature [121]. They found that the model had high sensitivity and specificity for their training data [121]. 

AI holds numerous other benefits; however, it also presents several limitations. The quality of AI’s output is contingent on the quality and quantity of input data, meaning the technology could be inherently biased due to skewed datasets originating from socioeconomic, racial, or geographic disparities in healthcare access [83]. Ethical and legal concerns about data privacy and algorithm transparency also exist [83]. Despite its potential, the current state of AI technology is not yet advanced enough to replace traditional preoperative planning techniques.

## 7. Conclusions

The history of preoperative imaging for breast reconstruction has been full of innovation. Throughout its evolution, it has remained inseparable from the progress made in our understanding of vascular anatomy and microsurgery. With the potential for further advancements on the horizon, particularly in the field of artificial intelligence, the continuous research and development in preoperative imaging techniques hold exciting promise for further enhancing the quality of breast reconstruction procedures and potentially improving the outcomes experienced by patients.

## Figures and Tables

**Table 1:** Advantages and disadvantages of CTA and MRA for preoperative planning in breast reconstruction.

	Computed Tomographic Angiography	Magnetic Resonance Angiography
Advantages	Can identify smaller-calibre vessels (down to 0.3 mm) Short imaging time High-resolution images Highly accurate perforator mapping Easy 3D reconstructions can be generated using post-processing software	No ionising radiation Can visualize blood flow dynamics High-resolution images Highly accurate perforator mapping Easy 3D reconstructions can be generated using post-processing software
Disadvantages	Exposure to ionising radiation Potential of allergic reaction to iodine-based contrast Less suitable for patients with renal insufficiency	Longer imaging time More expensive Potential of allergic reaction to gadolinium-based contrast Less suitable for patients with certain implanted medical devices (pacemakers, certain types of clips, etc.). Less suitable for claustrophobic patients

## Data Availability

Not applicable.
